# miRBase: annotating high confidence microRNAs using deep sequencing data

**DOI:** 10.1093/nar/gkt1181

**Published:** 2013-11-25

**Authors:** Ana Kozomara, Sam Griffiths-Jones

**Affiliations:** Faculty of Life Sciences, University of Manchester, Manchester, M13 9PT, UK

## Abstract

We describe an update of the miRBase database (http://www.mirbase.org/), the primary microRNA sequence repository. The latest miRBase release (v20, June 2013) contains 24 521 microRNA loci from 206 species, processed to produce 30 424 mature microRNA products. The rate of deposition of novel microRNAs and the number of researchers involved in their discovery continue to increase, driven largely by small RNA deep sequencing experiments. In the face of these increases, and a range of microRNA annotation methods and criteria, maintaining the quality of the microRNA sequence data set is a significant challenge. Here, we describe recent developments of the miRBase database to address this issue. In particular, we describe the collation and use of deep sequencing data sets to assign levels of confidence to miRBase entries. We now provide a high confidence subset of miRBase entries, based on the pattern of mapped reads. The high confidence microRNA data set is available alongside the complete microRNA collection at http://www.mirbase.org/. We also describe embedding microRNA-specific Wikipedia pages on the miRBase website to encourage the microRNA community to contribute and share textual and functional information.

## INTRODUCTION

miRBase is the public repository for all published microRNA sequences and associated annotation. miRBase was established in 2002, then called the MicroRNA Registry, with the primary aim of assigning stable and consistent names to newly discovered microRNAs. Novel microRNAs are submitted to miRBase after an article describing their identification is accepted for publication in a peer-reviewed journal. We aim to assign names quickly such that the official identifiers can be used in the final published version of the manuscript. miRBase microRNA gene names have the form dme-mir-100. The prefix signifies the organism, in this case *Drosophila melanogaster*. The numbers are assigned sequentially. Homologous microRNA loci in different species are assigned the same number. Paralogous microRNAs are assigned names with lettered and numbered suffixes, depending on whether the derived mature microRNA is identical in sequence, or contains sequence differences. The derived mature microRNAs were previously assigned names of the form dme-miR-100 and dme-miR-100*, for the guide and passenger strand, respectively. However, a growing body of evidence suggests that mature sequences derived from both arms of the hairpin may be biologically functional (1,2), and even that the dominant mature sequence can be processed from opposite arms in different developmental stages, tissues or between orthologous microRNAs (3,4). We therefore recently ceased use of the miR/miR* nomenclature in miRBase, in favour of assigning names of the form dme-miR-100-5p and dme-miR-100-3p for sequences derived from the 5′ and 3′ arms of the hairpin precursor. These naming guidelines are described in more detail in previous miRBase publications (5–8) and on the miRBase website and blog. It is important to note that the names are meant to be useful, but it is not possible (or desirable) to encode complex sequence relationships in a gene name. The name should therefore never be used as a substitute for rigorous sequence analysis.

miRBase distributes all published microRNA sequences, for browsing and searching by sequence and keywords, through a web interface (http://www.mirbase.org/), and for bulk download by FTP (ftp://mirbase.org/). The first release of miRBase in 2002 contained 218 microRNA loci from five species. Since then, the microRNA discovery field has exploded, with hundreds of microRNAs found to be present in each studied animal and plant genome. The number of published microRNA sequences in miRBase continues to increase rapidly, mainly driven by small RNA deep sequencing experiments. In the past 3 years, the number of microRNA loci annotated in miRBase has grown by approximately two-thirds, from 15 172 loci in 142 species (release 16, October 2010) to 24 521 loci in 206 species (release 20, June 2013). For each microRNA sequence entry, miRBase provides the primary references that describe its discovery, links to the evidence supporting the microRNA annotation, genomic coordinates and links to databases of predicted and validated microRNA target sites. Entries can be searched by sequence, keyword, literature reference and tissue expression.

Here, we describe two main areas of development of the miRBase database in the past 3 years. We have collected >300 publicly available small RNA deep sequencing data sets, and used patterns of mapped reads to assess the confidence in each microRNA annotation. We have also developed a system by which the microRNA community can contribute to textual and functional microRNA information using the Wikipedia resource.

## USING MULTIPLE DEEP SEQUENCING DATA SETS TO ANNOTATE HIGH CONFIDENCE MICRORNAS

For the past 5–6 years, the vast majority of novel microRNAs have been discovered by small RNA deep sequencing. These technologies have increased many fold the speed and ease with which novel microRNAs can be identified, published and submitted to a database. Increasing availability and decreasing cost of sequencing have also led to an increase in the number of research groups involved in microRNA discovery annotation. The rate of deposition of new microRNA sequences in miRBase therefore continues to increase. This growth represents a significant challenge to the overall quality of the microRNA sequence data set. As sequencing cost decreases, and depth of sequencing increases, researchers are annotating microRNAs that are expressed at lower and lower levels, or in more specific tissues, temporal stages or cell types. It therefore becomes more and more challenging to distinguish real microRNAs from fragments of other transcripts. Different research groups may use different stringencies of criteria to annotate loci as microRNAs, leading to variable quality data sets. A single poorly analysed data set has the potential to produce hundreds of bad microRNA sequences, swamping previous careful annotations. There have been several commentaries about the presence of dubious microRNA annotations in miRBase (9–12). Since its inception, miRBase has been run as a community resource. While we have a number of quality control measures, for example, to check submissions for fragments of ribosomal and transfer RNAs, the onus to demonstrate that a locus is a *bona fide* microRNA has remained with the submitting authors. The primary requirement for a novel microRNA sequence to be deposited in miRBase remains acceptance of a manuscript describing that sequence for publication in a peer-reviewed journal.

**Figure 1.**
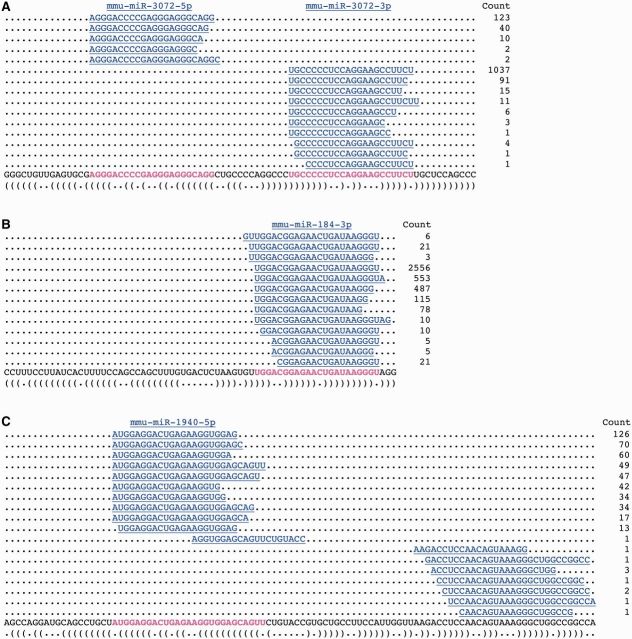
The patterns of small RNA deep sequencing reads mapping to three mouse microRNAs. Hairpin microRNA sequences are shown at the bottom of each panel, with derived mature microRNA sequences shown in magenta, and predicted base-paired secondary structure in dot-bracket notation underneath. Read sequences are shown in blue, with the summed count across all data sets on the right. (**A**) The annotation of mmu-mir-3072 is supported by reads mapped to both mature sequences, pairing with a 2-nt 3′ overhang, and mmu-mir-3072 is therefore annotated as a high confidence microRNA. (**B**) Reads from the available deep sequencing data sets map to only one arm of the mmu-mir-184 hairpin, which cannot therefore be annotated with high confidence. (**C**) The pattern of reads mapping to the annotated mmu-mir-1940 locus refutes the microRNA annotation—reads mapping to the two arms of the predicted hairpin do not pair with the 2-nt 3′ overhang characteristic of microRNA processing.

As previously discussed by us and others (5,12–14), the pattern of reads that map to a given hairpin locus can provide robust and powerful discrimination between a *bona fide* microRNA and other transcribed fragments. Large numbers of deep sequencing data sets are publicly available in databases such as the Gene Expression Omnibus (15) and the Short Read Archive (16), and these data sets allow us to assess *post hoc* the confidence in any given microRNA annotation. Since 2010, we have been collecting publicly available deep sequencing data sets from these two resources, and mapping reads to miRBase microRNA sequences (5). We currently collate 305 deep sequencing data sets from 38 species. From the patterns of reads mapped to a microRNA hairpin locus, we can often make one of three assertions: that the read pattern provides evidence for processing by Drosha and Dicer; that the read pattern is not consistent with microRNA processing; or that there are insufficient reads to provide adequate evidence for or against a microRNA annotation. Figure 1 shows an example of each of these three cases. In the case of mmu-mir-3072, multiple reads map to both arms of the predicted hairpin precursor. The most abundant read from each arm, representing the putative mature microRNAs, pair with a 2-nt 3′ overhang characteristic of processing by Drosha and Dicer. In contrast, the reads mapping to the mmu-mir-1940 locus are not consistent with microRNA processing. Although there are plenty of reads mapping to both arms of the predicted hairpin, they do not pair in a manner consistent with Drosha/Dicer processing. This annotation is likely to be incorrect; indeed, the sequence overlaps with an annotated H/ACA class small nucleolar RNA. This microRNA annotation should therefore be removed from future releases. However, for many microRNAs, we simply do not have enough read evidence to support or refute a microRNA annotation. For example, mmu-mir-184 has reads mapping to only one arm; this means it is much more difficult to determine from the reads alone whether the annotation is likely to be correct or not. We do not want to remove such microRNA entries from miRBase, but would like to distinguish the high and low confidence annotations.

We have been working on a system to use the available deep sequencing data sets to assign levels of confidence to microRNA annotations. Ideally this system should automatically assign a score or confidence level to each individual microRNA annotation. We would also like to allow manual curation to promote individual microRNAs into a high confidence set. In the first instance, we have focused on providing a high confidence subset of microRNA annotations across all species with available small RNAseq data sets in miRBase. To this end, we have assessed the pattern of deep sequencing reads that map to each microRNA annotation. To be annotated as high confidence, a locus must meet the following criteria:

- At least 10 reads must map with no mismatches to each of the two possible mature microRNAs derived from the hairpin precursor.
- The most abundant reads from each arm of the precursor must pair in the mature microRNA duplex with 0–4 nt overhang at their 3′ ends.
- At least 50% of reads mapping to each arm of the hairpin precursor must have the same 5′ end.
- The predicted hairpin structure must have a folding free energy of <−0.2 kcal/mol/nt.
- At least 60% of the bases in the mature sequences must be paired in the predicted hairpin structure.

**Figure 2.**
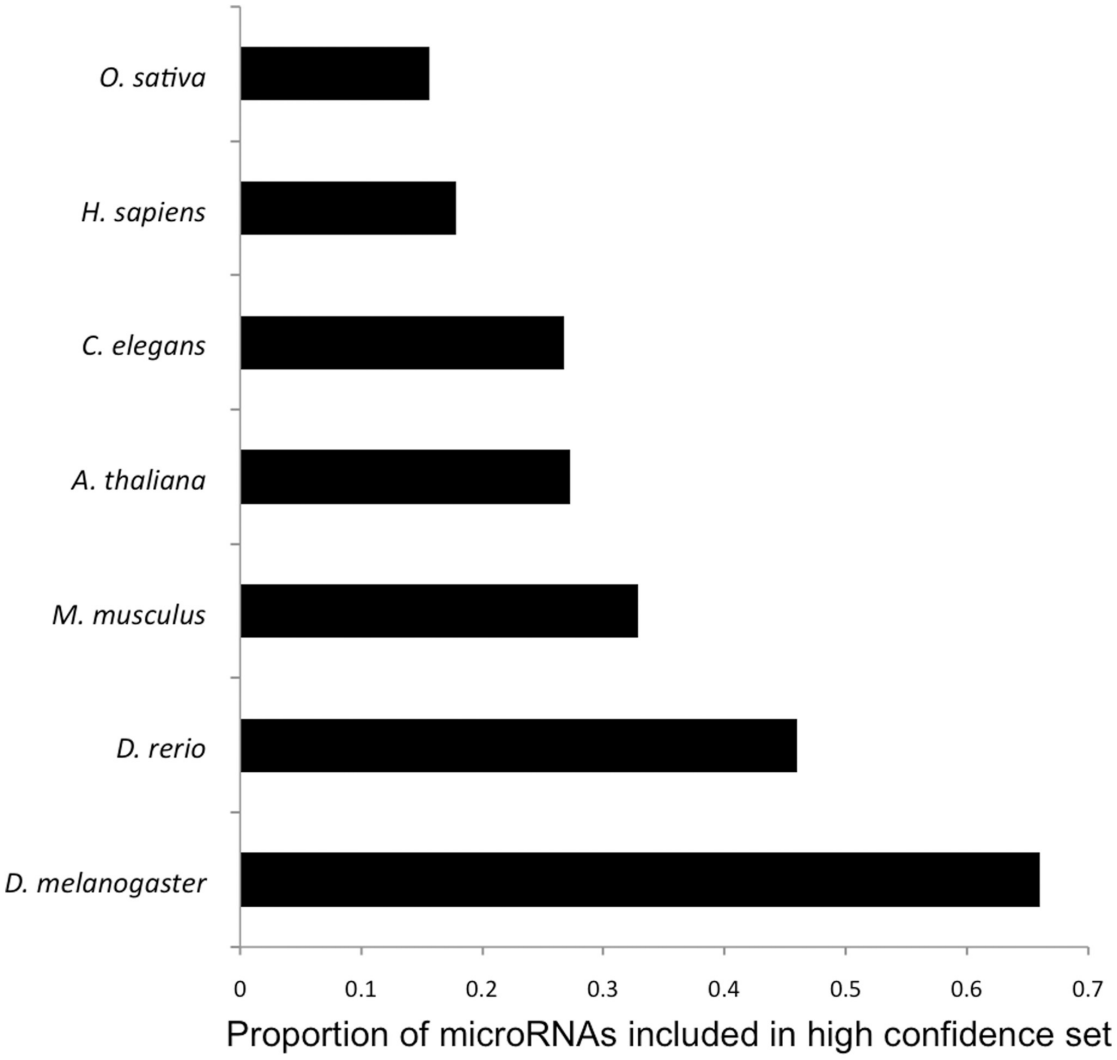
Proportions of microRNAs in model species that are included in the high confidence microRNA set.

We have applied these criteria to all microRNAs in the 38 species for which miRBase contains deep sequencing read data. In total, we annotate 1761 high confidence microRNA loci, representing 22% of the microRNAs in those 38 species. Figure 2 shows that different proportions of microRNAs from different organisms are classified as high confidence. It is important to re-iterate that the annotation confidence is assessed using the deep sequencing data sets that are collated in miRBase. We are actively collecting these data sets, but the current collection is more complete for some species than others. For example, miRBase has 50 individual small RNAseq data sets from *D. melanogaster*, representing >100 million reads. In addition, the *Drosophila* microRNA community has generally been conservative in their annotation of novel microRNAs. It is therefore not surprising that two-thirds of *D. melanogaster* microRNAs are annotated as high confidence. Most of the subset of sequences (60 of 73) not included in the high confidence set are omitted because there are not enough reads from miRBase data sets mapping to both arms of the hairpin precursor. The majority of these sequences were originally identified based on expression in RNAseq data sets that are not yet collected in miRBase.

In contrast, <20% of human microRNAs (278) currently pass all the criteria defined above. Again, in most cases, this is because the mature microRNAs are not represented by sufficient reads in the miRBase-collated deep sequencing data sets. Indeed, this criterion alone is responsible for the majority of microRNAs in all species that are omitted from the high confidence set. Of those microRNAs that are represented by sufficient reads to judge the processing patterns, the criterion that excludes most sequences is the requirement for consistent processing of the 5′ end of the mature sequences. This criterion has been discussed previously (5,13), and in our experience, reads with variable 5′ ends are a useful indicator of dubious microRNA annotations. However, several well-established microRNAs fail this test. For example, the human microRNA hsa-mir-126 is not automatically classified as high confidence because the reads mapping to both arms of the hairpin have somewhat variable 5′ ends. However, this microRNA otherwise shows the correct processing pattern, and has been extensively studied (17). This sequence is conserved in most vertebrates, and the mouse and zebrafish orthologues are included in the high confidence microRNA set. In such cases, it is clearly desirable to be able to manually promote a specific microRNA into the high confidence set. To facilitate this process, we have added buttons to ‘like’ and ‘dislike this miRNA’ to each microRNA entry page, along with an option to provide more information. Feedback from the community in this way will allow us to revisit and reassess specific microRNA annotations on demand.

It is important to note that non-canonical microRNAs may be expected to fail one or more of the criteria for high confidence. For example, the vertebrate microRNA mir-451 has been shown to be processed by a Dicer-independent pathway (18). Similarly, mirtrons are processed in a Drosha-independent manner by the splicing machinery (19–21). In some cases, these mechanisms may lead to significantly different read patterns from those expected of canonical microRNAs, and therefore non-canonical microRNAs are currently under-represented in the high confidence data set.

## COMMUNITY ANNOTATION

**Figure 3.**
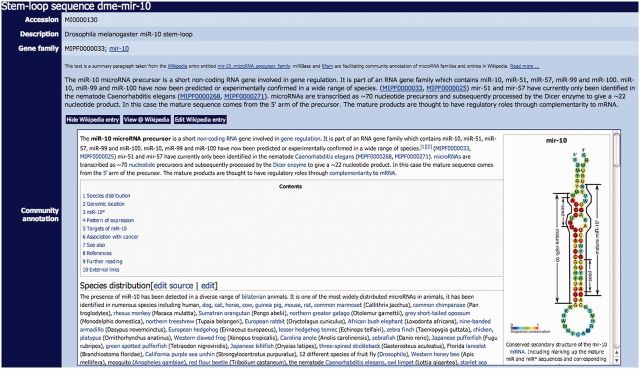
The miRBase entry page for dme-mir-10, showing the embedded Wikipedia page on the mir-10 microRNA precursor family.

Curating textual annotation of microRNA sequences is an enormous task. At the time of writing, the number of publications in PubMed that contain the word ‘microRNA’ in the title, keywords or abstract is >25 000, with >5600 published in the first 9 months of 2013 alone. These publications contain a wealth of functional information about individual microRNAs, which has been almost entirely missing from miRBase entries. In the future, we hope to be able to use computational text-mining methods to categorize microRNA-related articles, and to extract biological meaning from the literature. A second approach to this problem is to allow and encourage the microRNA community to contribute textual annotation and functional information about specific microRNA sequences and families. Several biological databases have used Wiki technologies to allow community annotation of sequences, either by establishing a bespoke Wiki resource or using the existing Wikipedia online encyclopedia (22–26). The Rfam database of RNA families has pioneered use of the Wikipedia resource for biological sequence annotation, with great success (24). Wikipedia already contains many pages about specific microRNA sequences and families. We have embedded Wikipedia information from these pages into the miRBase website (see Figure 3). Where an appropriate Wikipedia page exists, the miRBase page shows the summary paragraph, the full page and a link to edit the page at Wikipedia. All edits appear in Wikipedia immediately, and in miRBase within 24 h. As with all Wikipedia pages, the microRNA information can be edited by anyone. Over 4800 miRBase entry pages currently embed and link to Wikipedia pages, representing 20% of the miRBase database entries. In the 18 months since we first provided this function (April 2012), the embedded microRNA Wikipedia pages have been edited 383 times by 101 different users, and a handful of pages have been transformed from short stubs into full detailed articles. The miRBase blog maintains a list of microRNA Wikipedia pages that could be improved and updated. We hope that distributing this information in miRBase, and providing links to edit the pages, will encourage miRBase users and microRNA experts to contribute their knowledge in the form of Wikipedia edits and new pages.

## FUTURE DEVELOPMENTS

The high confidence microRNA data set is expected to become more comprehensive and more useful as we collate and map more small RNA deep sequencing data sets. We are currently prioritizing the addition of data sets for species that are not otherwise represented. However, we are grateful for suggestions of useful and comprehensive sequencing data sets in SRA and GEO that can be included in miRBase.

Currently, we make the high confidence microRNA data set available alongside the total miRBase microRNA collection, and high confidence microRNAs are clearly marked on the entry web pages. As miRBase’s coverage of the available small RNAseq data sets increases, we envisage that the high confidence set will become the default miRBase view. Lower confidence annotations, both historical and from new publications, will remain available, appropriately tagged as such. Where there are specific data to suggest that a specific microRNA is not a *bona fide* annotation (for example, if a mature microRNA annotation represents a fragment of another type of RNA, or the sequence read pattern clearly differs from that expected for Drosha/Dicer processing—see Figure 1C), that sequence will be removed from the miRBase database. This is consistent with the current procedure for removing bad microRNA entries.

In the future, we intend to use a range of existing microRNA prediction tools to score each annotation using the mapped reads. For example, miRDeep is a well-used tool to predict microRNAs from small RNAseq data sets, which returns a score for each putative microRNA annotation (14). We also envisage that it will be appropriate to assign multiple levels of confidence, as discussed previously (5), and allow a search for microRNAs based on user-defined thresholds for each of the criteria used to define the confidence levels.

## AVAILABILITY

miRBase is freely and publicly available under the Creative Commons Zero licence. All miRBase microRNA sequence data and annotation are accessible through the website (http://www.mirbase.org/) and are available for bulk download by FTP (ftp://mirbase.org/). We welcome feedback and questions on any aspect of the miRBase resource, and requests for microRNA name assignments, to mirbase@manchester.ac.uk.

## FUNDING

Biotechnology and Biological Sciences Research Council [BB/G022623/1]. Funding for open access charge: University of Manchester RCUK Open Access block grant.

*Conflict of interest statement*. None declared.
